# Role of Macroautophagy in Mammalian Male Reproductive Physiology

**DOI:** 10.3390/cells12091322

**Published:** 2023-05-05

**Authors:** Doaa Kirat, Ahmed Mohamed Alahwany, Ahmed Hamed Arisha, Adel Abdelkhalek, Taku Miyasho

**Affiliations:** 1 Department of Physiology, Faculty of Veterinary Medicine, Zagazig University, Zagazig 44519, Egypt; 2 Department of Animal Physiology and Biochemistry, Faculty of Veterinary Medicine, Badr University in Cairo (BUC), Cairo, Badr City 11829, Egypt; 3 Faculty of Veterinary Medicine, Badr University in Cairo (BUC), Cairo, Badr City 11829, Egypt; 4 Laboratory of Animal Biological Responses, Department of Veterinary Medicine, Rakuno Gakuen University, Ebetsu, Hokkaido 069-8501, Japan

**Keywords:** bulk autophagy, selective autophagy, testis, spermatogenesis, germ cells, Sertoli cells, Leydig cells, testosterone, steroidogenesis

## Abstract

Physiologically, autophagy is an evolutionarily conserved and self-degradative process in cells. Autophagy carries out normal physiological roles throughout mammalian life. Accumulating evidence shows autophagy as a mechanism for cellular growth, development, differentiation, survival, and homeostasis. In male reproductive systems, normal spermatogenesis and steroidogenesis need a balance between degradation and energy supply to preserve cellular metabolic homeostasis. The main process of autophagy includes the formation and maturation of the phagophore, autophagosome, and autolysosome. Autophagy is controlled by a group of autophagy-related genes that form the core machinery of autophagy. Three types of autophagy mechanisms have been discovered in mammalian cells: macroautophagy, microautophagy, and chaperone-mediated autophagy. Autophagy is classified as non-selective or selective. Non-selective macroautophagy randomly engulfs the cytoplasmic components in autophagosomes that are degraded by lysosomal enzymes. While selective macroautophagy precisely identifies and degrades a specific element, current findings have shown the novel functional roles of autophagy in male reproduction. It has been recognized that dysfunction in the autophagy process can be associated with male infertility. Overall, this review provides an overview of the cellular and molecular basics of autophagy and summarizes the latest findings on the key role of autophagy in mammalian male reproductive physiology.

## 1. Introduction

In 1963, the word autophagy was coined by the Belgian cytologist and biochemist Christian de Duve who was rewarded the Nobel Prize in Physiology or Medicine in 1974 for his discovery of lysosomes and peroxisomes. In the 1990s, Japanese biologist Yoshinori Ohsumi identified autophagy-related genes. In 2016, Ohsumi won the Nobel Prize in Physiology or Medicine for his discovery of the molecular mechanisms of autophagy. Ohsumi’s discoveries opened the way to recognize the fundamental significance of autophagy in many physiological processes. 

Autophagy, a lysosome-mediated intracellular degradation pathway, is an evolutionarily conserved mechanism in eukaryotes. Autophagy is a Greek word where “auto” means self and “phagy” means eating. This implies that autophagy is a process in which the cell eats its own components, similarly to cellular cannibalism. 

Autophagy plays fundamental roles in numerous physiological processes [1]. The physiological roles of autophagy are eliminating unnecessary cargoes, sequestering organelles, recycling cellular components, controlling organelle homeostasis, promoting cell survival, and providing required resources [2]. Autophagy is an intracellular degradation process in which unwanted cargoes, such as old or damaged organelles, and unneeded proteins are sequestrated into double-membrane vesicles called autophagosomes and subsequently delivered to the lysosomes for degradation by lysosomal hydrolases [3]. The macromolecular contents from this digestion are released back into the cytosol in order to be reused for cellular and tissue remodeling [3]. Therefore, the catabolic role of the autophagy pathway allows various cell types to maintain and control cellular homeostasis, renew the cells, and provide energy [4]. Consequently, the dysregulation and dysfunction of autophagy are implicated in various types of diseases, such as neurodegenerative diseases (Alzheimer’s, Huntington’s, and Parkinson’s disease) and tumorigenesis [5,6].

In mammalian species, there are three types of autophagy—macroautophagy, microautophagy, and chaperone-mediated autophagy—and all of them promote the proteolytic degradation of cytosolic components and cargoes in the lysosome and the reuse of synthesized macromolecules by the cell [7]. Different types of autophagy share the common feature of the lysosomal degradation of damaged proteins but differ in their mechanisms of delivering the substrate to the lysosome [8]. Macroautophagy depends on the formation of autophagosomes in order to transport cargo to the lysosome [9], while micro-autophagy involves the direct uptake of cargo via the invagination of the lysosomal membrane [10]. Meanwhile, chaperone-mediated autophagy-targeted proteins are translocated across the lysosomal membrane in a complex with chaperone proteins that are recognized by specific-receptor-lysosomal-associated membrane proteins [10,11]. Neither microautophagy nor chaperone-mediated autophagy involves autophagosome formation but instead depends on the degradation function of lysosomes.

Macroautophagy is the best-characterized type of autophagy and is generally referred to as “autophagy” [12]. Furthermore, according to the nutritional status, autophagy can be categorized into non-selective autophagy [13] during nutrient deprivation and selective autophagy [14] in non-starved conditions. Non-selective autophagy is known as a bulk degradation mechanism that is generated by nutrient deficiency [15]. This bulk autophagy helps when recycling building blocks to counteract the shortage of nutrients. Non-selective autophagy randomly engulfs cytoplasmic elements in the autophagosomes that are degraded by lysosomal enzymes. Furthermore, selective autophagy helps maintain intracellular homeostasis in non-starving cells by specifically targeting and destroying selective cargo such as damaged mitochondria, aggregated proteins, excess peroxisomes, etc. [16]. Table 1 summarizes the types of selective autophagy according to the cargo. 

The basis of the selectivity is that specific autophagy-related genes or other effectors can precisely distinguish receptors on cargo materials and subsequently induce autophagosome formation [17]. While certain cargo receptors directly attach to their cargoes, various other receptors recognize the poly-ubiquitin chains connected to the surface of cargoes for selective autophagy in mammalian cells [17].

The essential role of selective autophagy in cellular homeostasis was highlighted in numerous publications [17]. The dysfunction and dysregulation of selective autophagy are dangerous to cells and cause a distinct range of disorders, including the central nervous system [5], liver [18], cardiovascular system [19], immune system [20], kidney [21], oral diseases [22], and cancers [6].

Several findings have verified that, under physiological conditions, autophagy happens in a wide range of tissues and cells [4,7]. Recently, it has been documented that autophagy is related to the male reproductive process and acts as an important modulator of male fertility [4]. However, how autophagy mediates the crosstalk between germ, Sertoli, and Leydig cells to ensure proper spermatogenesis and steroidogenesis remains poorly understood. Therefore, the present review attempts to shed light and provide insight into recent advances in understanding the roles of non-selective and selective macroautophagy in mammalian male reproductive physiology.

## 2. Mammalian Autophagy Machinery and Autophagy-Related Genes

Briefly, the process of macroautophagy includes five stages: initiation, elongation, maturation, fusion, and degradation. In mammals, the process of autophagy initiates the formation of intracellular membrane-bounded organelles enriched in phosphatidylinositol 3-phosphate, known as omegasomes, that is dynamically connected to the endoplasmic reticulum [23]. The small cup-shaped membrane structure termed phagophore is de novo formed from omegasomes. After that, this phagophore undergoes nucleation followed by elongation to engulf cargoes and close to form autophagosomes [24,25]. Autophagy is terminated with the fusion of the autophagosome with the lysosome to form an autolysosome with the subsequent degradation of autolysosomal content by lysosomal hydrolases [26]. The resulting simple molecules, including free fatty acids, amino acids, and nucleotides, are recycled back to the cytosol by lysosomal permease and reused as an energy source by the cell [27].

Autophagy is a complex process that is regulated by a series of protein complexes and signaling pathways. In mammals, the core autophagy-related (ATG) genes and their protein products are generally classified into the ULK1 protein kinase complex [28], Vps34-beclin1 class III PI3-kinase complex [29,30], ATG9A transportation system [31,32], ATG12 conjugation system [33], and LC3 conjugation system [34]. Table 2 illustrates the components and role of autophagy complexes participating in the mammalian autophagy machinery.

Autophagy is induced by a wide range of stimulating signals such as nutrient deficiency (e.g., amino acids and glucose), growth factors deprivation (insulin and insulin-like growth factors), the depletion of cellular energy levels (ATP), extra- or intracellular stress (endoplasmic reticulum stress, hypoxia, and oxidative stress), and pathogenic infections [35].

Cells can create mechanisms to adapt their metabolism to the conditions of nutrient availability or metabolic stress in order to maintain cellular homeostasis. The master regulatory complex, the mammalian target of rapamycin (mTOR), is a key player that regulates the rate of anabolic and catabolic processes in response to nutrient availability [36]. In nutrient-rich conditions, mTOR is activated to enhance the anabolism by stimulating protein synthesis, nucleotide synthesis, glycolysis, lipogenesis, and mitochondrial biogenesis and, at the same time, suppress cellular catabolism via the inhibition of autophagy. On the contrary, the lack of growth factors and amino acids inhibits mTOR and thus stimulates protein breakdown via the catabolic pathway by inducing autophagy [36].

In addition, when cells are starved, the adenosine-monophosphate-activated protein kinase (AMPK) is activated. Autophagy is promoted by AMPK, which is a key energy sensor that regulates cellular metabolism to maintain energy homeostasis [37]. Therefore, mTOR and AMPK are considered major negative and positive regulators of autophagy, respectively [37].

**Figure 1:**
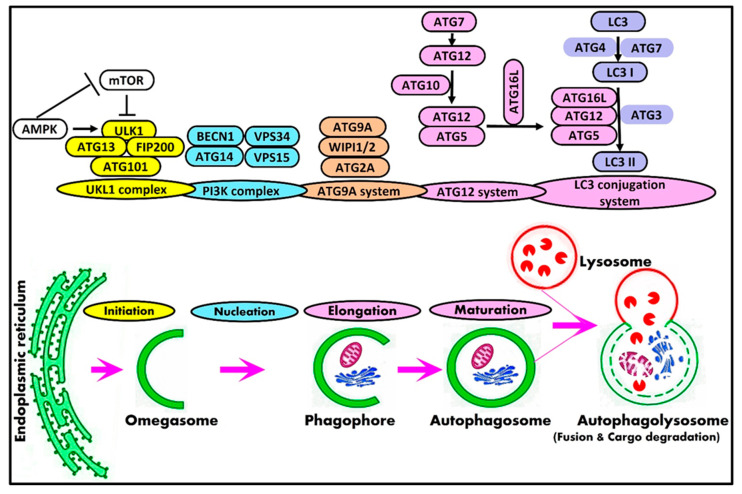
Schematic representation of macroautophagy pathway and core autophagy-related proteins in mammals.

A schematic representation of the mammalian autophagy machinery is shown in Figure 1. The core machinery of the initiation stage in mammalian cells is the Unc-51-like kinase (ULK) complex, which consists of ULK1/2, ATG13, FIP200, and ATG101 [38,39]. The dephosphorylation and autophosphorylation of ULK1, along with the dephosphorylation of ATG13, activate the entire autophagic cascade [40]. ULK1 post-translational modifications, such as phosphorylation [41] and ubiquitination [42], are essential for the induction of autophagy. Unphosphorylated ULK1 also promotes autophagosome–lysosome fusion [43]. AMPK and mTOR regulate autophagy via the direct phosphorylation of ULK1 [44]. In the initiation phase, the activation of AMPK by autophagy-stimulating signals inhibits mTORC1, which then dissociates from ULK1, leading to ULK1–AMPK interactions by which AMPK phosphorylates ULK1, activates ULK1 kinase, and dephosphorylates ATG13 in order to eventually initiate autophagy [3,44]. Additionally, the inhibited mTORC1 permits the ULK1 to phosphorylate ATG13, ATG101, and FIP200 [45], leading to the complete activation of the ULK1 complex. On the contrary, the activation of mTORC1 inhibits autophagy by inactivating ULK1/2 and ATG13 [44,46]. It has been shown that ATG101, the stabilizer of ATG13 [39], plays a crucial role in bridging the ULK1 and PI3K complex in mammalian autophagy induction [47].

Moreover, the process of nucleation is initiated when the activated ULK1 complex recruits the class III phosphatidylinositol 3-kinase (PI3K) complex, including VPS34, VPS15, Beclin1, and ATG14L, into phagophore initiation sites [48]. The activated ULK1 triggers the class III (PI3K) complex by phosphorylating beclin1 (BECN1) and vacuolar protein sorting 34 (VPS34) [3,49,50]. In the process of autophagy, the Beclin1-Vps34-Vps15-Atg14L complex is required for autophagosome nucleation and formation [3,31,51]. The activation of the Beclin1 complex generates phosphatidylinositol-3-phosphate, which is important for the nucleation of autophagosome [49]. Beclin1 interacts with VPS34, which activates VPS34 kinase activity to regulate the autophagosome’s size and quantity [29,49].

AMPK and ULK1 mediate the phosphorylation of ATG9A, which is required for proper ATG9A trafficking and autophagic flux [52]. The ATG9A transportation system consists of ATG9A, WIPI1/2, and ATG2A [32]. Phagophores require lipids and proteins to mature into autophagosomes. ATG9A is a lipid scramblase that has a key role in lipid mobilization from lipid droplets to autophagosomes for mediating autophagosomal membrane expansion and, hence, the progression of the autophagy process [53,54]. The ATG2 protein also transfers lipids, which are primarily needed for autophagosomal membrane expansion [55]. Both ATG2 and ATG9 are required for the expansion of the phagophore [56].

Moreover, an ULK1-independent ATG13-ATG101 complex regulates basal ATG9A trafficking [57]. During autophagy, WIPI1 and WIPI2 localize to autophagic membranes [58,59]. WIPI1 promotes the fission of endosomal transport carriers and the formation of autophagosomes. In autophagy initiation, WIPI1 binds omegasomes and enables the conjugation of LC3 to phosphatidylethanolamine (PE) in the LC3 lipidation process [60,61]. WIPI2 localizes to omegasome-anchored phagophores and upregulates LC3 lipidation [58].

Autophagosome elongation and maturation include two ubiquitin-like conjugation systems: the ATG12 conjugation system, the first ubiquitylation-like reaction, is essential for the formation and elongation of the autophagosome. The ATG12 conjugation system consists of ATG7, ATG5, ATG12, ATG10, and ATG16L1 [32]. ATG7 (E1-like enzyme) activates ATG12, and ATG12 is conjugated to ATG5 by ATG10 (E2-like enzyme) [62]. The ATG16L conjugates to ATG12-ATG5 to form the ATG12-ATG5-ATG16L complex, which promotes the elongation of the autophagic membrane and helps in the formation of the LC3-conjugated system [33,63].

The second ubiquitin-like conjugation system is the microtubule-associated protein 1 light chain 3 (LC3) conjugation system, and it consists of LC3, ATG7, ATG3, and ATG4. LC3 is widely used as an autophagosome marker in mammalian cells [64]. LC3 is cleaved by ATG4 to form LC3-I, which is then conjugated to phosphatidylethanolamine (PE) by ATG7 and ATG3 [65]. This reaction is catalyzed by the ATG12-ATG5-ATG16L complex [34]. PE-conjugated LC3 changed into a non-soluble form, which is LC3-II, and was steadily inserted into the autophagosome membrane [34]. LC3-II remains on mature autophagosomes until its fusion with lysosomes [66]. The number of LC3-II correlates to the number of autophagosomes [67]. As a result, the conversion of LC3 to LC3-II is considered a marker of autophagy induction.

The transport and fusion of autophagosomes with lysosomes are regulated by several molecules, such as Rab7 [68], EPG5 [69], SNAREs [70], LAMPs [71], FYCO1 [72], and PLEKHM1 [73].

Finally, the completion of the autophagy process requires degrading cargoes and transporting synthesized macromolecules back to the cytoplasm in order to be re-used for metabolic mechanisms and/or the synthetization of biomacromolecules [74,75].

### Potential Mechanisms of Selective Macroautophagy

The selectivity of autophagy is a prevalent phenomenon in various cells. Current research has proven the presence of various kinds of selective autophagy in eukaryotic cells. According to different cargoes, selective autophagy can be divided into several subcategories, such as mitophagy, proteaphagy, ribophagy, pexophagy, lysophagy, and nucleophagy [76] Table 1.

**Figure 2:**
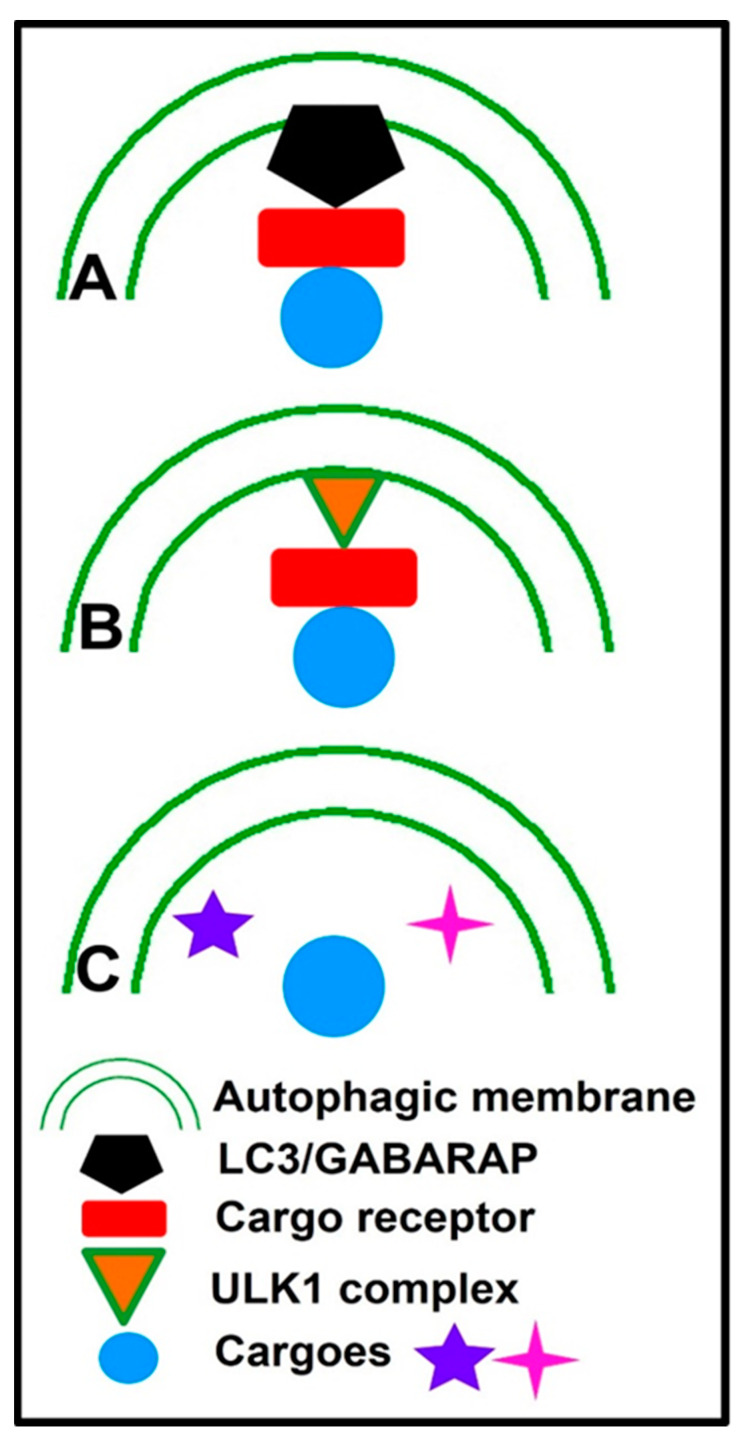
Representative overview of the mechanisms of selective macroautophagy.

Selective autophagy is principally dependent on both the recognition of the cargo and the coupling of the cargo to the phagophore, which can be carried out by proteins called selective autophagy receptors or cargo receptors [77]. A representative overview of the mechanisms of selective macroautophagy is illustrated in Figure 2. The first mechanism involves selective autophagy receptors that act as a bridge between the phagophore and cargo to facilitate the recruitment of autophagic machinery, mainly by the binding of LC3 and then the degradation of the cargo [78].

The second mechanism comprises the selection of cargo that can be achieved by targeted ubiquitination, which is recognized by ubiquitin-dependent selective autophagic cargo receptor proteins such as p62 [79,80], NBR1 [81], OPTN [82], and NDP52 [83], which in turn bind the cargo with ubiquitin to initiate pathways leading to autophagy initiation. Afterward, cargo is directed to the autophagosome by binding LC3/GABARAP proteins via the conserved LC3 interaction region and GABARAP-interacting motifs onto autophagic membranes [84]. The third mechanism for selective autophagy is that cargo receptors can recruit and bind the autophagy initiation ULK complex to induce autophagy. In mitophagy, the ULK complex is recruited to damaged mitochondria by cargo receptors OPTN and NDP52 [85]. The ER-phagy receptor CCPG1 (cell cycle progression 1) can bind to FIP200 as well as LC3 [86]. Moreover, selective cargoes, such as damaged organelles or ubiquitinated proteins, may accumulate at the autophagosome formation site and then are engulfed by autophagosomes even without direct recognition [87].

## 3. Physiological Relevance of Autophagy in Mammalian Tissues

Under physiological conditions, several studies verified that autophagy takes place and functions locally and specifically in a variety of mammalian tissues and organs. Autophagy not only plays crucial roles in the adaptive response relative to cellular stress (starvation, hypoxia, infection, and oxidative stress) but also in the maintenance of cellular homeostasis and quality control under normal physiological conditions [4,88].

Following various stresses, the autophagic machinery generates new building blocks and energy for anabolism to intensify cell function. Further, autophagy maintains cellular homeostasis in the absence of stress [88]. The homeostatic role of autophagy includes both non-selective degradation, which supports the basal turnover of cytoplasmic components, and selective degradation, which specifically targets damaged organelles or aggregated proteins [4]. Normal levels of autophagy play a vital role in the normal physiological activities of cells [43]. Therefore, defects in any step of the autophagy machinery process result in many diseases, including neurodegeneration, myopathy, diabetes, etc.

Evidence has revealed that autophagy acts in many tissues, such as the liver [89], pancreas [90], kidney [91], brain [92], skeletal muscle [93], heart [94], intestine [95], and placenta [96], as well as female [97] and male [98] reproductive systems.

In mammals, many studies have proved that autophagy has a beneficial role in health and disease prevention. Basal autophagy has cytoprotective roles in the maintenance of proper neuron and muscle function as well as in the homeostasis and survival of β cells [99]. Additionally, autophagy modulates innate and adaptative immunity [100]. Autophagy is also essential for many physiological processes and participates in the precise regulation of food intake, energy metabolism, lipolysis, glycogenolysis, gluconeogenesis, hepatokine secretion, cardiac mitochondrial homeostasis, insulin secretion, bone mass, osteoclast, osteocyte function, and muscle mass [101]. Table 3 summarizes the functions of autophagy in mammalian systems.

## 4. Macroautophagy and the Mammalian Male Reproductive Physiology

Autophagy affects several aspects of the male reproductive system. Research studies have revealed that autophagy has a vital role in differentiating spermatogonia into spermatozoa during spermatogenesis [124], acrosome biogenesis [125], testosterone biosynthesis [126], acrosome biogenesis [127], and flagella biogenesis [128] and ensuring Sertoli cell integrity [129]; moreover, it protects against the testicular damage caused by hyperglycemia [130] and hypoxia [131]. Defects in the autophagy process have been implicated in spermatogenesis impairment and male infertility, proving the prerequisite of autophagy homeostasis for normal spermatogenesis [132].

The function of the male reproductive system is to produce androgens such as testosterone as well as enhance spermatogenesis and transport into the female reproductive tract for fertilization. The testis is a reproductive gland that is part of the internal structures of the male reproductive tract. The specialized functional cells found in the testes primarily consist of Sertoli, Leydig, and different developmental stages of germ cells. Both types of testicular somatic cells, as well as germ cells, adopt autophagy as a regulatory mechanism for the maintenance of cellular homeostasis [102,125,127,133]. Under the physiological conditions of testicular tissue, the occurrence of autophagy results in maintaining the processes of spermatogenesis and steroidogenesis [124].

### 4.1. Autophagy and Male Germ Cell Development (Spermatogenesis)

The different germ cell types within the testicular seminiferous epithelium are spermatogonia, spermatocytes, spermatids, and spermatozoa. The process of the differentiation of a spermatogonium into a spermatozoon is known as spermatogenesis. Therefore, spermatogenesis is a complex cellular event that represents the entire process of germ cell development within the seminiferous epithelium of the adult testis. It consists of four distinct phases, namely, the (1) mitotic proliferation and differentiation of spermatogonia; (2) the meiotic division of spermatocytes; (3) spermiogenesis process, which leads to the transformation of haploid round spermatids to elongated spermatids; and the (4) spermiation process that causes the release of mature sperm into the lumen of seminiferous tubules [134].

Normal spermatogenesis demands an equilibrium between the degradation and energy supply to preserve the metabolic homeostasis of cells. According to recent reports, under physiological conditions, autophagy might contribute to different steps of spermatogenesis and plays an important role in male reproductive physiology. As a result, any impairment in the autophagy process can be associated with male infertility.

### 4.2. Autophagy and Spermatocytogenesis

Spermatocytogenesis is the initial phase of spermatogenesis. During this phase, spermatogonial stem cells (SSCs) at the basal membrane of the seminiferous tubule proliferate by mitotic division to produce identical spermatogonia for balancing self-renewal or differentiating into two diploid primary spermatocytes [135]. The imbalances in SCC self-renewal and differentiation before meiosis result in impaired spermatogenesis as well as male infertility [136].

In mammalian spermatogenesis, studies have been previously focused on the role of autophagy in somatic cells and spermatids but not SSCs, which might be critical for the maintenance of the SSC pool and subsequent meiosis. Recently, the dynamic expression patterns of autophagy-related genes in spermatogonia, the late stage of primary spermatocytes, and the early stage of spermatids were observed by [137].

Various classical autophagic-related genes were found to exist, and they are actively expressed in human spermatogonia (AKT1, ATG5, EPG5, and TBC1D20), spermatocytes (PRKACA, ATG7, SIRT1, and RARA), and spermatids (TSC1) [132]. Moreover, Wang et al. [132] have recently proved that the newly defined autophagic gene, Cst3, plays a critical role in the maintenance of mouse spermatogonia stem cells (mSSCs) and the subsequent development of male germ cells by regulating the autophagy process in human and mice. Cst3, which is actively expressed in spermatogonia and early spermatocytes, was found to regulate SSC maintenance. In humans and mice, the Cst3 autophagic gene was highly expressed in SSCs and throughout differentiation from spermatogonia to early spermatocytes, and HSPD1 was also expressed in spermatogonia and leptotene spermatocytes, while DRAM1 was highly expressed in late spermatocytes and round spermatids [132]. Furthermore, Cst3-knockdown mSSCs exhibited an increase in LC3a expression when compared to that in control SSCs. Transmission electron microscopy also revealed the apparent accumulation of autophagosomes in Cst3-knockdown SSCs. In addition, autophagic inhibitors such as 3-Methyladenine and chloroquine significantly inhibited Cst3-knockdown-induced autophagy initiation in mouse SSCs. Accordingly, autophagy is triggered by Cst3 knockdown. Sirtuin 1 (SIRT1; a member of the mammalian sirtuin gene family) modulates the autophagy process, which is necessary for male fertility by participating in the differentiation of spermatogenic stem cells during spermatogenesis in mice [127]. Consequently, autophagy homeostasis is important for spermatogonia stem cell maintenance and normal spermatogenesis, as well as male fertility.

As a master regulator of cellular metabolism, mTOR is predominantly expressed in human spermatogonia and late-stage spermatocytes [132]. mTORC1 has vital roles in meiotic progression, the maintenance of the SSC pool, and the silencing of sex chromosomes in the male mouse germline [138,139]. Other reports have also indicated the requirement for mTORC1 signaling in the normal proliferation and differentiation of undifferentiated progenitor spermatogonia in rats [140,141] and humans [142,143]. In germ cell conditional knockout mice for the mTORC1-specific component Raptor, SSC proliferation showed a reduction in the neonatal testis and blockage in juvenile and adult testes [139]. Consequently, this induces infertility due to meiotic arrest and the impaired inactivation of sex chromosomes in the male germline [138]. The rapamycin analog, everolimus, inhibits mTOR signaling with the subsequent inhibition of spermatogonial differentiation in juvenile and adult mouse testes [144]. Retinoic acid (RA), as a necessary driver of spermatogonial differentiation and meiosis, activates the PI3K-AKT-mTOR signaling network to stimulate receptor tyrosine kinase (KIT) expression, which is required for spermatogonial differentiation in mouse testes [145]. The overexpression of Lin28a, a marker of SSCs [146], promotes the self-renewal and proliferation of SSCs via the activation of PI3K/AKT and mTOR in goats [147].

### 4.3. Autophagy and Spermiogenesis

The last phase of spermatogenesis is spermiogenesis, which is considered a highly orchestrated developmental process. Spermiogenesis represents the post-meiotic male germ cell differentiation stage, in which the haploid round spermatid is differentiated into an elongating mature spermatozoon in the seminiferous epithelium right before its release into the lumen of the seminiferous tubule [148]. The major events in this phase include the remodeling of the nucleus’s shape, the condensation of chromatin content, the formation of the acrosome, the removal of excess cytoplasm/organelles, the reorganization of mitochondria, and the development of the sperm tail [148,149].

Autophagy is substantially involved in the spermiogenesis process as it regulates male haploid round and elongating spermatids; hence, the impairment of autophagy leads to various spermatozoa defects, such as the formation of an abnormal sperm head, coil sperm tail, and sperm aggregation [125,128,150]. Based on the current literature, some autophagy-related proteins have been reported to play important roles in spermiogenesis.

Human sperm have the molecular machinery necessary for autophagy activation [151]. Proteins related to autophagy, such as ATG5, LC3, ATG16, BECN1, AMPKα, and mTOR, are present and functionally active in human spermatozoa, suggesting their involvement in the regulation of sperm survival and motility [151]. Recently, growing evidence demonstrated that the autophagy genes ATG7, LC3, and Beclin-1 are expressed and localized in normal human testicular tissue [152]. Furthermore, previous results have shown that both LC3-I and LC3-II are present in stallion spermatozoa and associated with sperm survival [153,154]. Aparicio et al. demonstrated that autophagy marker LC3 is activated and regulates sperm viability during equine semen cryopreservation [155]. ATG7 was also required for the removal of extra cytoplasm during mice [128] and rat [150] spermiogenesis. Yang et al. proved that in vivo autophagy developed within male haploid germ cells as elongation progressed [150]. The expression of LC3 could be observed firstly in round spermatids in the testis of a 20-day-old mouse, which indicates the involvement of autophagy in early testicular spermatid development [128]. The immunolocalization of LC3 and ATG7 showed marked increases in their expression in the male haploid round to elongated spermatids as spermiogenesis progresses from the basal toward the lumen of rat seminiferous tubules [150]. In addition, these authors detected, by using transmission electron microscopy, increased numbers of autophagosomes and lysosomes in elongated spermatids as spermiogenesis progressed in rat testis. ATG5 was found to be required for normal spermiogenesis and male fertility by maintaining normal autophagy functions in mouse germ cells [133]. AMPK, a positive autophagy regulator [44], is located on the midpiece of mammalian sperm [156]. AMPK is essential for the motility of the sperm and the integrity of sperm membranes [157,158].

Cytoplasm organelle removal is important for the formation of mature sperm in order for sperm to be viable for fertilization [159]. Lei et al. recognized the expression of armadillo repeat-containing 3 (ARMC3) in mice testicular tissues; the expression is important for the autophagic elimination of cytosolic ribosomes during spermiogenesis and hence gives energy for flagellar motility [160]. The deficiency of ARMC3 results in male infertility and the failure of ribosome removal in elongated spermatids [160].

Furthermore, it was reported that the decreased gene expression levels of LC3b, Beclin1, and ATG5 and increased BCL-2 expression might possibly correlate with the inhibition of the autophagy process in azoospermic patients [161]. ATG5 or ATG7 knockout mice showed spermatozoa with reduced motility and malformed heads [162]. The sperm motility of germ cell ATG7 knockout mice dropped significantly and retained some cytoplasm on the head of mature sperm and impaired the cytoskeleton’s organization [128]. The inactivation of sperm AMPK affects boar sperm motility [157]. Recently, Huang et al. [133] verified that the conditional ATG5-deficient male mice exhibited reduced fertility with decreased sperm numbers and abnormal sperm morphology, including aberrant acrosome formation.

Autophagy not only has a role in eliminating pre-existing materials but also supports the subsequent production of new components [7]. Autophagy also regulates the biogenesis of acrosomes [125,128,133]. In spermatozoa, the acrosome is a testis-specific organelle required for male fertility. It is a unique organelle that covers the anterior part of the sperm nucleus and plays a vital role in the process of fertilization. The acrosome reaction is one of the most critical steps in fertilization [163]. Autophagy marker proteins ATG9 and LC3 were localized by immunocytochemistry on the acrosome and the equatorial segment of male sperm [164]. It was demonstrated that defects in the autophagy process by knocking out the ATG7 gene in germ cells led to infertility with respect to abnormal acrosome formation, which resulted in round-headed sperm, proving the importance of autophagy and the requirement of ATG7 in acrosome biogenesis during spermatogenesis in mice [125,128]. Moreover, TBC1 domain family member 20 (TBC1D20), the key regulator of autophagosome maturation, is required for the formation of acrosomes in mouse testes [165]. Moreover, Sirtuin 1 is involved in acrosome biogenesis by modulating autophagic flux during spermiogenesis in mice [127]. Additionally, it was recently elucidated that tudor domain-containing 7 (TDRD7) regulates the maturation of autophagosomes and hence plays an essential role in acrosome biogenesis in mice [166].

Indeed, autophagy is also crucial for flagella biogenesis in spermatids during spermiogenesis. Shang et al. [128] revealed that the reduction in the motility of ATG7-null spermatids is principally due to the loss of the proper “9 + 2” microtubule structures in the axonemes of aggregated sperm flagella during spermiogenesis in mice.

Altogether, autophagy participates in the shaping of the head, the removal of excess cytoplasm, acrosome biogenesis, and flagella assembly during spermiogenesis in elongating spermatids [167]. An alteration in the expression of autophagy pathway genes may be associated with male infertility. Hence, the germ-cell-specific disruption of autophagy-related genes impaired the autophagic flux, leading to defects in acrosome biogenesis, retention of excess cytoplasm, decreased sperm motility, and decreased testicular weight in mice [125,133,168].

### 4.4. Autophagy and Spermiation

At the end of spermatogenesis, elongated spermatids are released from Sertoli cells into the lumen of the seminiferous tubule by a process called spermiation. Spermiation involves several discrete steps, including the removal of excess cytoplasm from around the spermatid flagella, the extension of the spermatid into the lumen of seminiferous the tubule, the progressive removal of specialized adhesion structures, the formation and degradation of tubulobulbar complexes, and the disengagement of the spermatid from the Sertoli cell into the lumen of the seminiferous tubule [169].

In mammalian testes, tubulobulbar complexes are actin-filament-based structures that form at the intercellular junctions in the seminiferous epithelium. They comprise blind-ended tubular projections that extend into Sertoli cells from junctions with adjacent cells [170]. These complexes take place at the junctions between Sertoli cells and mature spermatids in the apical region of seminiferous tubules as well as at the junction between neighboring Sertoli cells near the basal region of seminiferous tubules [171]. Tubulobulbar structures act as cellular attachment structures that ensure sperm attachment to Sertoli cells before their release. A major goal of spermiation is to degrade the apical ectoplasmic specialization junction to facilitate the release the spermatozoa into the lumen [172]. Therefore, the impaired degradation of tubulobulbar complexes is associated with spermiation failure [170].

It is known that autophagy maintains the cytoskeletal organization of certain structures [173]. Autophagy is needed for the degradation of ectoplasmic specialization and tubulobulbar complex components in Sertoli cells [162]. Autophagy is found to be active near tubulobulbar complex regions. Recently, studies by Wang et al. [137] observed that the immunolocalization of autophagic markers LC3, ATG5, and ATG7 was visualized adjacent to the component of tubulobulbar complexes and that the LC3 signal increased in tubulobulbar complexes isolated from spermatids attached with Sertoli cell regions.

It has been recently demonstrated that suppressed autophagy in the Sertoli cells of ATG7-deficient mice and ATG5-deficient mice showed the accumulation of some tubulobulbar components around the spermatid head, which subsequently results in failure in the spermiation process [137]. Moreover, autophagy disruption in Sertoli cells causes the accumulation of negative cytoskeleton organization regulator PDLIM1 (PDZ and LIM domain protein 1) and thus leads to defects in the assembly of apical ectoplasmic specialization in Sertoli-cell-specific ATG5 or ATG7-deficient mice [162]. Overall, the degradation of apical ectoplasmic specialization and many tubulobulbar complex components via the autophagy–lysosome pathway in Sertoli cells is required for spermiation.

## 5. Autophagy and Sertoli Cells

Autophagy physiologically occurs in mammalian Sertoli cells [174,175]. Sertoli cells are testicular somatic cells that are present in the seminiferous tubules of males [176]. They are one of the most important cells necessary for supporting spermatogenesis. Sertoli cells are recognized as ‘nurse cells’ that are responsible for providing nutritional requirements as well as structural and energy support to developing germ cells [177].

Moreover, Sertoli cells have pivotal roles in the autocrine and/or paracrine regulation of spermatogenesis. Sertoli cells have receptors for FSH and testosterone, which enable them to be the major targets for testosterone and FSH signals that regulate spermatogenesis and male fertility [178]. FSH upregulates Sertoli cell proliferation by activating the PI3K/Akt/mTORC1 complex signaling pathway in the cultured proliferating Sertoli cells of rats [179]. mTOR expression in Sertoli cells is essential for the maintenance of spermatogenesis and the progression of germ cell development via the pachytene spermatocyte stage [180]. mTOR regulates Gap Junction Alpha-1 (GJA1) protein distributions in Sertoli cells and is needed for the maintenance of spermatogenesis in mice [180]. In vivo functional verification for the significance of mTOR signaling in testicular postnatal Sertoli cells was elucidated by inhibiting its expression in a transgenic mouse model. The inactivation of mTOR in Sertoli cells results in testicular atrophy, the disorganization of the seminiferous epithelium, a loss of Sertoli cell polarity, increased germ cell apoptosis, the premature release of germ cells, decreased epididymal sperm counts, increased sperm abnormalities, and infertility [180]. This failure in the capability to maintain spermatogenesis is probably caused by the improper trafficking of GJA1 [180].

It was observed that the deficiency of Raptor in Sertoli cells displays severe tubular degeneration in the neonatal mouse testis as well as azoospermia in adult mice [181]. More recently, [182] proved that Raptor is required to maintain Sertoli cell identity, stabilize the male pathway, and promote testes development in mice.

Autophagic markers ATG7 and LC3 were found to be immunolocalized in the Sertoli cells of young and adult goats [183]. In mammals, serine/threonine protein kinase ULK1/2 plays key regulatory functions in the initiation of autophagy [184]. The knockdown of ULK1 in goat Sertoli cells was shown to inhibit the autophagy process as approved by the decreased expression of autophagic markers LC3, Beclin1, ATG5, and ATG7, leading to a decline in both Sertoli cell viability and the gene expressions of goat Sertoli cell markers (ABP, AMH, FASL, and GATA4) [185]. In addition, the knockdown of the ULK2 gene inhibits autophagy in swine Sertoli cells [186].

Rubicon, a negative regulator of autophagy, was controlled by androgens to prevent the autophagic degradation of GATA4, which is a transcription factor needed for Sertoli cell function [187]. It has been shown that the genetic loss of Rubicon in Sertoli cells was associated with the upregulation of autophagy and caused defective spermatogenesis in mouse testes [187].

The results by Liu et al. [162] revealed that the disruption of autophagy in Sertoli cells gives rise to the disorganization of seminiferous tubules, the disruption of ectoplasmic specialization, and the production of sperm with deformed heads due to the accumulation of PDLIM1. Therefore, autophagy can protect the function and integrity of Sertoli cells by inhibiting the disruption of ectoplasmic specialization [129].

Additionally, Sertoli cells secrete a variety of vital molecules, including the anti-Müllerian hormone, androgen-binding protein, inhibin, and activin [175]. These secretions facilitate spermatogenesis either directly or indirectly by using a hormonal negative feedback system. The androgen-binding protein can increase the concentration of testosterone in the seminiferous tubule to promote spermatogenesis [175]. Autophagy regulates the expression level of the androgen-binding protein in rat Sertoli cells. The results of knockdown experiments with small interfering RNA in rat primary Sertoli cells showed that the inhibition of autophagy by ATG7 siRNA enhanced the expression of androgen-binding proteins, while the stimulation of autophagy by mTOR siRNA decreased its expression [175]. It has also been reported that LC3, the most common autophagic-related protein [188], colocalized with the androgen-binding protein in primary rat Sertoli cells, implying that autophagy degrades and clears the androgen-binding protein in rat Sertoli cells [175]. Furthermore, this clearance is regulated by testosterone. Testosterone inhibits autophagy and upregulates the expression of androgen-binding proteins in rat Sertoli cells [175].

## 6. Autophagy and Leydig Cell Steroidogenesis

Testosterone is the major androgen in the testis that regulates spermatogenesis and is essential for male fertility. Testosterone is produced by the Leydig cell, the major cell type that populates the interstitial regions of the testis, in response to stimulation with luteinizing hormones [189].

In mammals, autophagy is extremely active in Leydig cells. The relative frequency of autophagy in Leydig cells is greater than that in other cell types [190]. Autophagosomes were visualized in the Leydig cells of rats [191], whereas autophagosomes and autolysosomes were observed in the Leydig cells of mouse testis [98].

It was demonstrated that impaired autophagy in Leydig cells is linked with the reduction in the serum levels of testosterone in rats, mice, and humans [102,126,192,193,194,195]. It has been shown that the reduction in testosterone production is correlated with decreased autophagic activities in the Leydig cells of aged rats. The knockdown of Beclin 1 in Leydig cell lines derived from mouse testes leads to a decrease in the expression of steroidogenic acute regulatory (StAR) protein and testosterone production [126]. Additionally, there was a decrease in the levels of sex hormones in ATG5-null mice [195]. Results by Gao et al. [102] confirmed that autophagy disruption by the conditional knockout of ATG7 or ATG5 in steroidogenic cells induces a sharp decline in serum testosterone levels and consequently affects the sexual behavior of male aging mice. The LC3 protein localizes at the Leydig cells of mouse testis [98]. Moreover, impaired autophagy was detected in low-testosterone patients as the expression of the autophagic marker; LC3 significantly declined in the Leydig cells from patients exhibiting azoospermia or oligospermia with low serum testosterone levels [102].

Furthermore, in vivo and in vitro findings by Chen et al. [196] confirmed that autophagy activity is connected to steroidogenesis in the Leydig cells of dairy goats. The Leydig cells of dairy goats treated with an autophagy inhibitor (3-methyladenine; 3-MA) showed a decrease in testosterone production, while their treatment with an autophagy activator (rapamycin) resulted in the enhancement of testosterone production [196].

Recently, it has been shown that N6-methyladenosine (m6A) mRNA methylation regulates testosterone synthesis by modulating autophagy in Leydig cells [196]. m6A, the predominant internal modification in mRNA, regulates murine spermatogenesis [197]. It is involved in germline development as it modulates protein synthesis in spermatogonia stem cells and in spermatids [197]. Further studies revealed that m6A modification affected AMPK activity by promoting the translation of PPM1A (protein phosphatase 1A, magnesium-dependent, and alpha isoform), the negative AMPK regulator, whereas it decreased the expression of calcium/calmodulin-dependent protein kinase 2 (CAMKK2) beta, the positive AMPK regulator, by reducing its mRNA stability, resulting in reduced AMPK activity and autophagy inhibition with subsequent suppression in testosterone synthesis in Leydig cells [198].

Interestingly, autophagy regulates testosterone synthesis by facilitating cholesterol uptake in Leydig cells. Activated steroidogenic cells demand high levels of cholesterol substrates. Cholesterol acts as a substrate for testosterone biosynthesis in Leydig cells [199]. The uptake of cholesteryl esters is selectively mediated by the class B type I (SR-BI) scavenger receptor in steroidogenic cells [200]. Na^+^/H^+^ exchanger regulatory factor (NHERF2) is a protein binding partner for SR-BI that negatively regulates the expression of the SR-BI protein via the inhibition of its de novo synthesis [201]. Gao et al. [102] provided evidence that autophagy in Leydig cells regulates SR-BI via the enhancement of NHERF2 degradation; thus, autophagy could facilitate and enhance cholesterol uptake into Leydig cells during testosterone synthesis.

Similarly, SIRT1 controls the synthesis of testosterone in Leydig cells by regulating the autophagy process [202]. SIRT1 is a vital regulator of autophagy via the direct deacetylation of ATG7 and LC3, thereby facilitating their translocation from the nucleus to the cytoplasm [203]. The level of lysosome-associated membrane protein 2 (LAMP2), a marker for the presence of lysosomes or autolysosomes, has also been affected by the absence of SIRT1 [204]. In the Leydig cells of SIRT1 knockout mice, an increase in LC3 acetylation and a decrease in LAMP2 levels were obviously detected [202]. Furthermore, NHERF2 seems to act as a link between SIRT1 and cholesterol absorption in testicular steroidogenic cells. It was found that the disruption of SIRT1 in mouse Leydig cells significantly increased NHERF2, which consequently hinders the cholesterol uptake in Leydig cells via SR-BI downregulation [202]. Therefore, these recent findings by Khawar et al. [202] revealed that SIRT1 disruption in Leydig cells impairs autophagy, which in turn results in a clear decrease in testosterone levels and interrupts steroidogenic activity in male mice.

Collectively, all these findings add weight to prove the concept that autophagy might function as an important regulator for testosterone biosynthesis to support spermatogenesis in mammals.

## 7. Autophagy and Fertilization

Spermatozoa progress through many maturational stages throughout their journey, beginning from their production in the testes and ending with oocyte fusion, allowing them to perform fertilization. After their release into the epididymis, immotile testicular sperm progress through further physiological and biochemical modifications, referred to as epididymal maturation [205]. To gain their fertilizing capacity, sperm must undergo two sequential extra-testicular maturational events: epididymal maturation in the male tract and capacitation in the female tract prior to penetration into the oocyte [206,207].

Until recently, in the mammalian epididymis, the gene expression of autophagy-related molecules has been examined only in bison and pigs. Many factors, such as mTOR, ULK1, ATG13, PI3K, beclin1, beclin2, ATG14, ATG5, ATG16L, and LC3, were detected in the epididymal tissues of adult male European bison [208], while Beclin1, LC3, mTOR, ATG12, and ULK1 were expressed and visualized in the epididymis of Congjiang Xiang pigs [209].

Capacitation is a unique feature of mammalian sperm. It results in various changes in sperm, involving hyperactive motility, the activation of some signaling pathways and, essentially, the ability to undergo the acrosome reaction [210]. Capacitation is accompanied by modifications in the lipid composition of the plasma membrane and posttranslational changes in proteins regulated by several signaling pathways [211].

The proper physiological levels of reactive oxygen species (ROS) are vital for normal spermatozoa and their surrounding environment [212]. Mammalian spermatozoa are the active production site of ROS through the NADPH oxidase system at the level of the spermatozoa plasma membrane and the reduced nicotinamide adenine dinucleotide (NADH)-dependent oxidoreductase system at the level of mitochondria [213,214]. ROS, such as superoxide anion, nitric oxide, and hydrogen peroxide, act as signaling molecules by triggering intracellular pathways that are required for activating several processes in sperm, such as maturation, hyperactivation, capacitation, chemotaxis, and acrosome reactions [215]. Several studies have recognized the physiological role of ROS in potentiating the capacitation process [216]. Under physiological conditions, a low concentration of ROS produced by spermatozoa is required for the activation of signal transduction processes associated with capacitation [216]. ROS are crucial mostly at the beginning of capacitation as regulators of cholesterol efflux, the activation of the cAMP/PKA pathway, tyrosine phosphorylation, or redox signaling [217].

Conversely, overproduced levels of ROS have severe pathological impacts on the sperm, varying from reduced sperm concentration and motility to decreased fertilization. Sperm cells are highly susceptible to ROS-induced damage. Therefore, it is critical for the development of fully mature spermatozoa to balance the processes involved in the formation and degradation of ROS in order to achieve the required male fertilization capacity [218].

It has been reviewed that ROS can regulate autophagy, and in turn, autophagy can regulate oxidative stress [219]. It is now widely accepted that the accumulation of ROS induces cellular autophagy, which acts to reduce ROS levels and oxidative damage to maintain cellular homeostasis [220,221,222]. Nevertheless, the exact pathway describing how autophagy regulates the extreme production of ROS is still unknown.

ROS could initiate autophagosome formation and autophagic degradation. Increased ROS levels can regulate autophagy via the oxidation of ATG4 [223]. Oxidized ATG4 prevents the ATG4-mediated cleavage of LC3-II/PE, leading to autophagosome formation and, hence, maintaining autophagy as the active state [223]. Moreover, it has been reviewed that ROS regulates autophagy via transcriptional and post-transcriptional regulations [224].

ROS can initiate autophagy via the activation of AMPK and the inactivation of mTOR. In this context, starvation-induced autophagy involves the ROS-mediated activation of AMPK [222]. ROS may also induce autophagy/mitophagy via the downregulation of the PI3K/AKT/mTOR signaling pathway [225]. Recently, Jin et al. [226] showed that the reduction in autophagy induced by peroxiredoxin 2 (Prx2; a member of the peroxidase family of antioxidant enzymes) was correlated with the inhibition of ROS in N2a-APP Swedish cells.

Similarly to capacitation, sperm chemotaxis depends on the production of ROS [227]. ROS have been found to facilitate chemotactic movements of the spermatozoa towards the oocyte in the female reproductive tract against a concentration gradient of progesterone secretion by cumulus oophorus cells [227]. Only capacitated spermatozoa are chemotactic [228]. Results by Imai et al. [229] proved that the trafficking of ATG9A via the recycling endosomes is important for autophagosome formation. This autophagy marker (ATG9) is mostly found in the head of sperm in both normal and cryptorchid spermatozoa [164]. Recently, it has been revealed that ATG9A ubiquitination regulates oxidative-stress-induced autophagy [230]. Moreover, Campisi et al. [231] revealed that the ATG9A protein contributes to the chemotactic movement of various cell lines. To date, some G-protein-coupled receptors (GPCRs) have been recognized in mammalian sperm and are implicated in sperm motility and acrosome reactions [232]. A link between GPCR signaling and the autophagy machinery has been detected. Chemotactic GPCRs control cell migration by inhibiting autophagosome biogenesis [233].

The acrosome reaction constitutes the last maturational stage of the spermatozoon in order to be able to carry out fertilization. It involves modifications of the anterior part of the spermatozoa head and the release of acrosomal enzymes following contact with the zona pellucida of the oocyte in order to allow the sperm to penetrate the zona pellucida and fuse with the oocyte membrane [234].

Autophagy is involved in proacrosomal vesicle fusion and transport to form the acrosome. Because of the deformed acrosome during autophagy disruption, it is expected that the acrosomal reaction could be impaired. The PFN3 and PFN4 members of the profilin gene family are detected in the testes and play roles in autophagy regulation during acrosome biogenesis [235,236]. PFN3 is expressed in the acroplaxome–manchette complex of the developing sperm, the Golgi complex, and proacrosomal vesicles during spermiogenesis, implying a role in vesicle transport for acrosome biosynthesis [235]. In PFN3-deficient mice, the activation of mTOR and the inhibition of AMPK cause the suppression of autophagy flux, which is characterized by the accumulation of LC3B [235]. Similarly, PFN4 was found to be localized in the acrosome–acroplaxome–manchette complex, and it is essential for acrosome biogenesis and manchette development during mouse spermiogenesis [236]. More recently, Umer et al. [236] showed that failure in the biogenesis of acrosome in PFN4-deficient mice is due to increased levels of PI3K, AKT, and mTOR and reduced levels of AMPK, which result in the blockage of autophagic flux during spermiogenesis. Consequently, autophagy disruption either in PFN3-deficient or PFN4-deficient mice leads to impairment in acrosome biogenesis and subsequently marked reductions in acrosome reactions [235,236].

## 8. Selective Macroautophagy and the Mammalian Male Reproductive Physiology

### 8.1. Mitophagy

Although mitochondria can be engulfed non-selectively along with other cytosolic contents during bulk autophagy, mammalian cells can selectively degrade damaged or superfluous mitochondria by mitophagy [237,238,239]. Mitophagy is a conserved cellular process that is crucial for maintaining normal cellular physiology. It contributes to the mitochondrial quality control mechanism as it selectively eliminates damaged and unhealthy mitochondria, thereby maintaining the quality of the organelle [240]. It also mediates the degradation of paternal mitochondria during embryonic development. Accordingly, mitochondrial dysfunctions due to mitophagy deficiency are implicated in many diseases.

**Figure 3:**
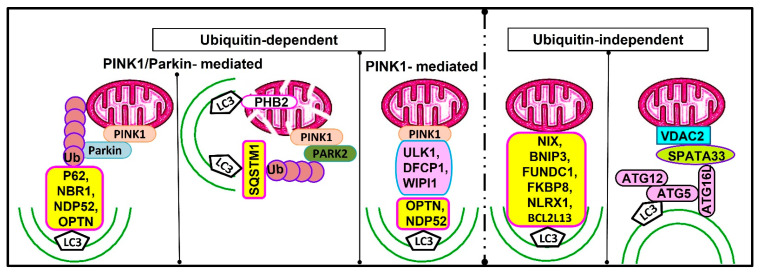
Schematic presentation of mitophagy mechanism and regulation in mammals.

In mammals, mitophagy has several mechanisms, including ubiquitin-dependent (either PINK1/Parkin-dependent or PINK1-dependent mitophagy) as well as ubiquitin-independent pathways (Figure 3).

In the PINK1/Parkin-mediated pathway, upon mitochondrial impairment or the loss of mitochondrial potential, PINK1 accumulates on the outer membrane of damaged mitochondria [241], leading to the autophosphorylation of PINK1 and then the phosphorylation of ubiquitin [242], which could further bind and recruit Parkin to the outer membrane of damaged mitochondria. The activated PINK1 phosphorylates Parkin and triggers its ubiquitin ligase activity [243]. The activated PARKIN ubiquitinates some outer mitochondrial membrane proteins and produces polyubiquitin chains that are identified by autophagic receptors OPTN, P62, NBR1, and NDP52, which subsequently interact with the phagophore-inserted LC3 to engulf mitochondria in the autophagosome and become degraded by the lysosome [244].

Further, in the Parkin-independent pathway, the activated PINK1 ubiquitin kinase is sufficient for recruiting autophagy receptors OPTN and NDP52, which in turn recruit ULK1, DFCP1, and WIPI1 into the mitochondria and subsequently bind to LC3 to directly induce mitophagy independent of Parkin [85].

The third mechanism for mitophagy is the ubiquitin-independent pathway in which mitochondrial outer membrane receptor proteins, such as NIX, BNIP3, FUNDC1, BCL2L13, FKBP8, and NLRX1, contain a conserved LC3-interacting receptor (LIR) domain, which directly recognizes and binds to LC3 to initiate the selective clearance of mitochondria [245].

In the male reproductive tract, mitochondria are the key components of energy conversion and metabolism in sperm. It is more than just a powerhouse of the cell. Mitochondria are multitasking organelles and contribute to a variety of physiological processes, such as spermatogenesis, sperm functions, and male fertility. Their participation has been implicated in the differentiation of spermatogonial stem cells, meiotic processes, cellular differentiation in round spermatids, testicular somatic cell development, the structural organization of sperm tail, energy production for mature sperm motility, sperm quality, hyperactivation, sperm capacitation, acrosome reaction, ROS homeostasis, fertilization, testicular somatic cell development, and the biosynthesis of testosterone [246,247,248].

Mitochondria are structurally and functionally unique organelles in male gametes. They are localized in the middle piece of the spermatozoa [246]. During spermiogenesis, while much of the cytoplasm containing mitochondria is lost, there are around 72–80 remaining mitochondria arranged around the midpiece of the sperm tail [246]. The morphology and number of mitochondria in male germ cells are modified according to the differentiation status of cells during spermatogenesis [249]. The OXPHOS forms of mitochondria were found in spermatogonia and early spermatocytes, whereas late spermatocytes, spermatids, and sperm harbor more condensed, elongated, and metabolically efficient forms [250].

Selective autophagy has been documented to relate to reproductive processes, especially for spermatogenesis, fertilization, and biosynthesis of testosterone. Mitophagy plays an essential role in the process of sperm differentiation before fertilization and the removal of paternal mitochondria after fertilization. During spermiogenesis, the removal of excess mitochondria by mitophagy is essential to produce individual sperm with proper mitochondrial rearrangements [133,251]. Mitophagy regulates sperm motility and viability in humans [151]. At the molecular level, it has been demonstrated that deletions and other changes to mitochondria DNA result in lowered sperm functionality and male infertility in humans [252] and mice [253]. In humans, impaired sperm mitochondria are associated with a decrease in sperm motility, high ROS production, and infertility [246,254]. It has been reported that the disruption of mitochondrial rearrangements in male germ cell conditional knockout ATG7 mice was due to mislocalized and badly condensed mitochondria [128]. Similarly, ATG5 knockout male germ cells showed abnormal sperm differentiation with failure in mitochondrial rearrangements during spermiogenesis because of impaired autophagy [133].

During spermatogenesis, ubiquitination has roles in cell signaling and gene transcription regulation [255]. The ubiquitin-dependent mechanism for the recognition and elimination of defective spermatozoa was demonstrated in the mammalian epididymis [256]. Spermatids are ubiquitinated during spermatogenesis in rats and mice [257,258]. Ubiquitin is present on the surface of defective human spermatozoa [259].

The ubiquitin tag of sperm mitochondria is acquired during spermatogenesis in order to promote the degradation of defective sperm in the epididymis as well as sperm mitochondria after fertilization [260]. Spermatocyte mitochondria are already ubiquitinated, and most mitochondria are lost to the discarded residual body during spermiation. In bulls, mitochondrial ubiquitination is detected at the secondary spermatocyte phase, the round spermatid, and mature spermatozoa [261]. The mitochondria of mouse sperm are also ubiquitinated [262].

Extensive studies indicate that the maternal inheritance of mitochondrial DNA relies on the selective elimination of sperm-derived paternal mitochondria during early embryogenesis [263]. There are several mechanisms explaining how entire sperm mitochondria are degraded inside fertilized mammalian oocytes. Sperm mitophagy describes the recognition and degradation of sperm mitochondria. In mammals, the autophagic pathway and ubiquitin–proteasome system contribute to sperm mitophagy after fertilization [264]. Sutovsky et al. [262] reported the ubiquitination of sperm mitochondria in fertilized eggs from rhesus monkeys and cows. Moreover, the mitochondria of mouse sperm exhibited immunopositivity with respect to p62, LC3, and GABARAP after fertilization [260].

Post-fertilization sperm mitophagy relies on ubiquitin-binding autophagy receptor sequestosome 1 (SQSTM1) in the zygotes of boars and rhesus monkeys. SQSTM1 was detected in the midpiece/mitochondrial sheath of sperm tails in the fertilized oocytes of porcine and rhesus monkeys, while it was absent from sperm until fertilization [264]. Therefore, the SQSTM1 in sperm mitochondria originated from the oocyte cytoplasm and is involved in the recognition of sperm mitochondrial proteins in the mitophagy pathway in boars and rhesus monkey zygotes [264]. Moreover, the ubiquitination of paternal mitochondrial proteins and its presentation to the 26S proteasome by the proteasome-interacting ubiquitinated dislocase protein and valosin-containing protein (VCP) could be strictly required as determinants of sperm mitophagy [264].

In synergy with SQSTM1-regulated mitophagy, PARKIN contributes to post-fertilization sperm mitophagy in mouse zygotes [265]. Mitochondrial E3 ubiquitin protein ligase 1 (MUL1) acts similarly to the PINK1/PARKIN pathway in ubiquitination and mitophagy processes [265]. It was found that PARKIN and MUL1 synergistically function in mitochondrial degradation in mouse zygotes, and the depletion of one of them decreased paternal mitochondrial degradation [265].

Other candidate sperm-borne mitophagy determinants in the spermatozoa are spermatogenesis-associated 18 (SPATA18) and PARKIN co-regulated gene (PACRG). SPATA18 expression is an essential player in the mitophagy process. The SPATA18 protein is present in satellite fibers that are associated with dense outer fibers in the middle piece of human sperm, and it is important for sperm motility and fertility as the sperm motility level is directly proportional to SPATA18 expression [266].

The existing concept is that the ubiquitinated mitochondrial outer membrane is the primary target of engulfment by autophagosomes during mitophagy [242]. However, Wei et al. [267] identified a mitochondrial inner membrane protein named prohibitin 2. It is one of the components of the mammalian sperm-mitochondrial inner membrane that undergoes ubiquitination during spermatogenesis [268]. Prohibitin is localized in the sperm mitochondrial sheath as well as in the mitochondria of the elongated and round spermatids of bulls and rhesus monkeys [268]. These authors also proved that prohibitin is ubiquitinated in sperm mitochondria, spermatids, and the testicular spermatozoa of bulls. The ubiquitination of prohibitin and other substrates may occur in defective spermatozoa during the epididymal passage.

Thompson et al. [268] demonstrated that there are two distinct pathways for the ubiquitination of sperm mitochondria according to sperm status (normal or defective). In normal sperm, prohibitin is ubiquitinated mainly in the testis and masked by disulfide bond cross-linking during the epididymal passage. After fertilization, these ubiquitin–prohibitin complexes are subsequently unmasked to serve as a signal on which the oocyte’s proteolytic degradation system specifically targets the sperm mitochondria. While in defective sperm, prohibitin and other mitochondrial membrane proteins are further ubiquitinated to ensure their degradation during the epididymal passage.

Prohibitin 2 is a key mitophagy receptor for Parkin-mediated mitophagy in mammalian cells. Prohibitin 2 binds to LC3 during mitophagy via the LC3-interacting region (LIR) motif. The binding of prohibitin 2 to LC3 requires the prior rupture of the outer mitochondrial membrane [267]. This can be attained via the PARK2-mediated ubiquitination of outer mitochondrial membrane proteins followed by the recruitment of the proteasome to mitochondria, the degradation of outer mitochondrial membrane proteins, and the rupture of the outer mitochondrial membrane [269]. The resulting rupture of the outer membrane allows LC3-II on a phagophore to bind mitochondrial inner membrane prohibitin 2, leading to mitophagy (Figure 3).

Recently, [270] identified SPATA33 as a novel autophagy mediator for mitophagy, specifically in male germlines, and it could play important roles in spermatogenesis. SPATA33 is expressed in spermatogonia, spermatocytes, round spermatids, and the spermatozoa of mice [270]. The mitophagy process via SPATA33 is probably ubiquitin-independent and does not interact with autophagic machinery LC3 [270]. It has been demonstrated that the SPATA33 protein localizes on mitochondria via the interaction of its C-terminus with the mitochondrial outer membrane protein VDAC2 [270]. To initiate mitophagy, ATG16L1 binds to the N-terminus of SPATA33 to recruit and sequester the damaged mitochondria by autophagosomes during starvation. SPATA33 is a mediator linking VDAC2 and ATG16L1 [270].

### 8.2. Lipophagy

Lipophagy is a ubiquitous pathway responsible for the autophagic degradation of intracellular lipid droplets [32]. It is the main player involved in the breakdown of triglycerides and cholesterol esters stored in lipid droplets and the subsequent release of free fatty acids and free cholesterol in a wide range of cell types and tissues [32]. The disruption of this lipophagic balance has been implicated in human health and disease. Either the activation or suppression of lipophagy plays a central role in tissue physiology [107,271,272,273,274].

**Figure 4:**
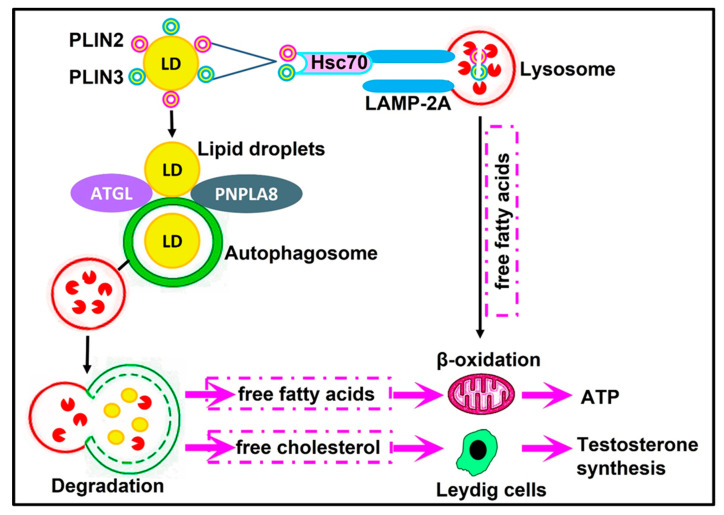
Overview of lipophagy mechanism and regulation in mammals.

Lipophagy has an important role in the regulation of cellular energy homeostasis and lipid homeostasis [17]. Lipid droplets are selectively sequestered by the autophagosome and degraded by hydrolase after fusion with the lysosome to generate free fatty acids [275]. A schematic overview of lipophagy mechanisms and regulation in mammals is shown in Figure 4.

Lipid droplet’s coat proteins: Perilipin 2 (PLIN2) and perilipin 3 (PLIN3) are degraded by chaperone-mediated autophagy to allow lipophagy receptors to access lipid droplets and hence facilitate lipophagy [276]. The lipophagy receptors in mammals, adipose triglyceride lipase (ATGL) and patatin-like phospholipase domain containing 8 (PNPLA8), recognize lipid droplets, leading to the promotion of its catabolism and the subsequent oxidation of hydrolyzed free fatty acids to maintain cellular energy homeostasis [17]. In contrast, ATG14, which contains a PE-biding region, can interact with LC3 and ULK1 to initiate lipophagy, which results in a continuous supply of free fatty acids to maintain sufficient levels of mitochondrial β-oxidation in order to provide energy [17]. Therefore, lipophagy allows the cell to rapidly adapt to nutrient deficiency by producing ATP from the increased oxidation of free fatty acids.

Lipophagy has the potential to participate in steroid production and spermatogenesis regulation. Lipid homeostasis is crucial for testosterone secretion in Leydig cells [277]. Steroidogenesis in Leydig cells is a vital process that is indirectly linked with spermatogenesis. Testosterone is required for critical steps during spermatogenesis, such as the maintenance of the blood testes barrier, meiosis, Sertoli–spermatid adhesion, and sperm release [189]. In steroidogenic cells, the utilization of cholesterol-containing lipid droplets is important for the production of several steroid hormones [278]. Studies on the importance of autophagy and lipophagy in Leydig cell steroidogenesis have been reported in naked male mole-rats, rats, and mice [102,103,202,279]. Lipophagy modulates the degradation of cholesteryl esters to generate free cholesterol, which is a substrate of testosterone synthesis [102,103,202]. Autophagy-deficient Leydig cells showed dramatic reductions in lipid droplets, cholesterol, and testosterone synthesis, which may ultimately influence spermatogenesis and fertility [102,103,202,279].

### 8.3. ER-Phagy

The endoplasmic reticulum is a highly dynamic intracellular organelle that synthesizes, modifies, folds, and transports proteins. The homeostasis of the endoplasmic reticulum is vital for cell survival. The disruption of this homeostasis by a variety of stimuli leads to endoplasmic reticulum dysfunction due to the accumulation of unfolded and misfolded proteins in its lumen and the subsequent initiation of endoplasmic reticulum stress [280]. Various physiological conditions associated with increased protein demands result in enhanced levels of unfolded and misfolded proteins that accumulate in the lumen of the endoplasmic reticulum.

Once the cells face any stimuli, various protective mechanisms can be stimulated to resume the homeostasis of these cells. In mammals, when endoplasmic reticulum stress occurs, two pathways are triggered to cope with this stress and resume/restore the function of the endoplasmic reticulum [281]. The first pathway is the unfolded protein response (UPR) via the activation of three signal pathways of endoplasmic reticulum transmembrane proteins, including protein kinase R-like ER kinase (PERK), inositol-requiring protein 1 (IRE1), and activating transcription factor (ATF6), whereas the second pathway is endoplasmic reticulum-associated degradation (ERAD), which facilitates the transport of misfolded proteins back to the cytosol in order to be degraded via the ubiquitin–proteasome system [281].

However, when endoplasmic reticulum stress is intense or persists for a time, both UPR and the ubiquitin–proteasome system are still insufficient to allow the endoplasmic reticulum to be restored to its normal state. In such cases, endoplasmic reticulum stress will initiate protective ER-phagy under physiological conditions. It has been shown that endoplasmic reticulum stress negatively regulates the AKT/TSC/mTOR pathway to enhance autophagy [282].

**Figure 5:**
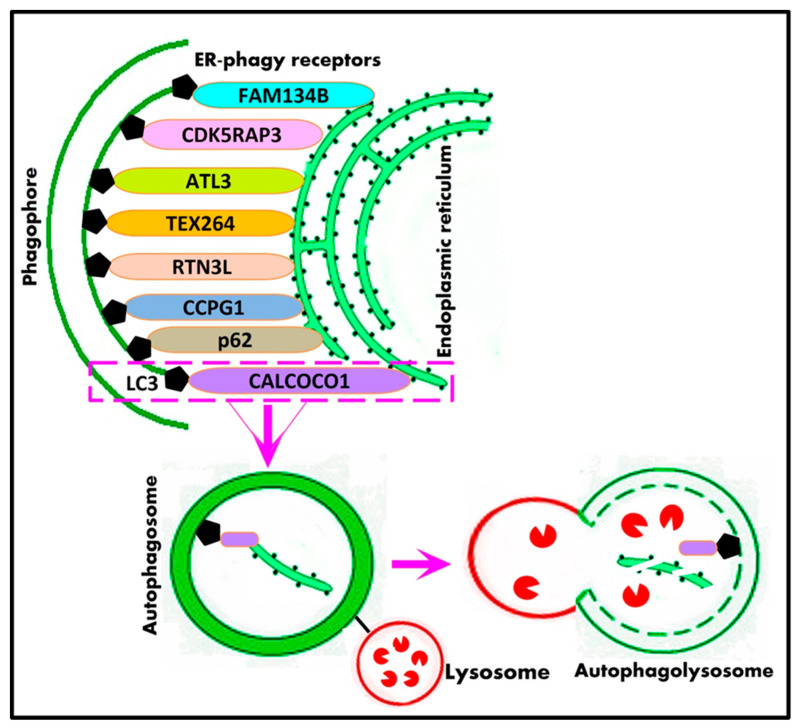
Schematic overview of ER-phagy mechanism in mammals.

ER-phagy is a form of selective autophagy that involves an autophagosome that directly connects to the endoplasmic reticulum via ER-phagy receptors that can bind with the LC3 through its LC3-interacting (LIR) domains [283,284]. This was followed by the subsequent degradation of the damaged endoplasmic reticulum into small fragments that reassembled into a new endoplasmic reticulum, which restored its normal state. Consequently, ER-phagy seems to be the last opportunity to restore endoplasmic reticulum homeostasis [285]. Thus, ER-phagy results in the clearance of excessive endoplasmic reticulum in a time-dependent manner to provide stable mammalian cells. In mammals, FAM134B, FAM134A, FAM134C, RTN3L, CCPG1, TEX264, ATL3, CALCOCO1, p62, and CDK5RAP3 have recently been identified as receptors for macro-ER-phagy [286]. A schematic overview of ER-phagy mechanisms in mammals is illustrated in Figure 5.

Spermatogenesis requires massive protein synthesis for the differentiation of spermatogonia to spermatozoa throughout mitosis and meiosis in the testis. Endoplasmic reticulum stress might be a new signaling pathway for regulating ER-phagy in male reproduction. With respect to endoplasmic reticulum stress, the Grp78 chaperone protein was strongly detected throughout the stages of pachytene spermatocytes in murine and human testes [287]. Additionally, Lachance et al. [288] confirmed the localization of Grp78 in spermatocytes and round spermatids as well as in the neck region of ejaculated sperm in the human testis. These results indicate that in addition to being expressed in human testis spermatogenic cells, Grp78 proteins persist in ejaculated spermatozoa. Thereby, the participation of Grp78 in spermatogenesis and sperm functions during fertilization suggests an important function for endoplasmic reticulum stress signaling pathways in the process of spermatogenesis [287,288].

Infertility could be a consequence of impairing the endoplasmic reticulum stress response in male reproduction [289]. Aging in men is linked with a decrease in the level of testosterone because of the degeneration of Leydig cells [290]. It has been demonstrated that excessive endoplasmic reticulum stress is closely linked with aging-related diseases and male reproductive dysfunction [291]. Results by Huang et al. [292] reported that oxidative stress, as well as the endoplasmic reticulum stress signaling pathway, have essential roles in the degenerative changes in Leydig cells in aging mice. Evidence has revealed that hypoxia downregulates androgen-biosynthesizing genes, such as steroidogenic acute regulatory protein (StAR) and 3-β-hydroxysteroid dehydrogenase (3β-HSD), in the testis of rats by increasing calcium influx, oxidative stress, and the upregulation of endoplasmic reticulum stress signaling molecules Grp78, PERK, and CHOP [293,294].

Mature spermatozoa do not have endoplasmic reticulum. Nevertheless, resident chaperones on the endoplasmic reticulum, such as ER protein 29 (ERp29) and calreticulin, can be transferred to the spermatozoa to perform significant functions in sperm fertilization and acrosome reactions [295,296]. In the mammalian reproductive tract, endoplasmic reticulum chaperones were shown to be important for spermatogenesis as well as for post-testicular sperm maturation in the epididymis and female reproductive tract [296]. ERp29 is localized on the spermatozoa in the epididymal caput, caudal regions, and on acrosome-reacted spermatozoa in mouse testes, implying its possible involvement in sperm fertilization by facilitating sperm–oocyte membrane fusion [295].

Calreticulin is a unique ER luminal resident protein that affects many cellular functions both inside and outside the ER. It has key roles in chaperoning as well as in the regulation of Ca^2+^ homeostasis [297]. Calreticulin is not specific to testes, but it is closely linked with spermatogenesis in rats [298]. Calreticulin is predominantly expressed in the acrosome of both round spermatids and the mature spermatozoa of rats [298], bulls [299], and humans [300]. Nevertheless, it was visualized in cytoplasmic droplets and the midpiece region of human spermatozoa [300], and the principal piece of bull sperm flagella [299]. Consequently, calreticulin is considered a major candidate in the regulation of calcium oscillation patterns during acrosome reactions and sperm hyperactivation.

Recently, calreticulin has been identified as a novel regulator of ER stress via the calreticulin–autophagy axis [301]. It has been demonstrated that calreticulin expression is stimulated by the UPR in response to ER stress. Calreticulin subsequently induces the formation of autophagosomes and initiates autophagic flux. The LIR motif of calreticulin becomes linked with LC3 in the autophagic machinery to inhibit ER stress via the degradation of misfolded proteins [301].

## 9. Conclusions

Autophagy is key to maintaining cellular homeostasis. As illustrated by collective research on the latest literature discussed in this review, the normal functions of male reproductive systems require basal autophagy for maintaining homeostasis. Autophagy adjusts the function of the male reproductive system by bulk and selective degradation. Autophagy has a beneficial role in maintaining integrity, health, and the normal physiology of mammalian male reproductive systems. Basal autophagy has physiological functional roles in the maintenance of proper spermatogenesis, sperm survival and function, and testicular steroidogenesis, as well as in recycling different cellular nutrients to maintain energy homeostasis. Thereby, an impaired autophagy process adversely affects male reproductive functioning, hence inducing male infertility. It could be possible to therapeutically modulate autophagy to harness its benefit for the treatment of autophagy-associated male infertility.

## 10. Future Perspectives

### Oxidative Stress–Autophagy Axis and Male Fertility

Homeostatic imbalances in the reproductive system of males influence spermatogenesis as well as Leydig cell steroidogenesis and even lead to infertility. Male infertility is a global health problem. Oxidative stress is one of the most important causes of male infertility [302]. It is capable of disrupting both the capacity of the germinal epithelium to differentiate normal spermatozoa and the steroidogenic capacity of Leydig cells [303]. The mutual regulation and restriction of oxidative stress and autophagy guarantee the balance of physiological homeostasis in male reproductive organs. The interplay of autophagy and oxidative stress has yet to be fully elucidated in the reproductive system of males. It has been demonstrated that ROS both promotes and impairs autophagy. ROS have both beneficial and destructive effects on sperm functions depending on their levels. Physiological concentrations of ROS have crucial roles in the activation of normal sperm physiological processes, such as sperm maturation, capacitation, hyperactivation, acrosome reactions, chemotactic processes, and fusion with the female gamete, to ensure fertilization. However, the excessive generation of ROS results in a state of oxidative stress that can cause infertility by impairing sperm quality and disrupting the fertilizing capacity of spermatozoa and the structural integrity of their DNA. Oxidative stress principally results from lifestyle-related issues. In particular, oxidative stress principally results from the individual’s lifestyle, and ROS can be produced due to heat stress, smoking, aging, obesity, insulin resistance, psychological stress, and chronic strenuous exercises [302]. Therefore, understanding the fine molecular regulation of autophagy by ROS, as well as the tight relationship between the metabolism and redox state, is required in order to provide strategies that can be plausible therapeutic approaches for overcoming the burden of oxidative-stress-induced male infertility.

## Figures and Tables

**Table 1:** Types of selective autophagy and their corresponding cargo.

Selective Autophagy	Cargo
Mitophagy	Mitochondria
Lipophagy	Lipid droplets
ER-phagy	Endoplasmic reticulum
Glycophagy	Glycogen
Ribophagy	Ribosomes
Lysophagy	Lysosomes
Myelinophagy	Myelin
Zymophagy	Zymogen granules
Pexophagy	Peroxisomes
Aggrephagy	Protein aggregates
Ferritinophagy	Ferritin
Xenophagy	Bacteria and viruses

**Table 2:** Components and role of autophagy complexes participating in the mammalian autophagy machinery.

Complex	Core Components	Autophagic Role
ULK1/2 complex	ULK1/2 ATG13 FIP200 ATG101	Initiation
BECN1 complex	Beclin1 VPS34 VPS15 ATG14L	Nucleation
ATG9A complex	ATG9A WIPI1/2 ATG2A	Initiation
Ubiquitin-like complex	LC3A-C, GABARAP	Cargo selection and Elongation
	ATG12 ATG4 ATG7 ATG3 ATG10 ATG5 ATG16L1	Elongation

**Table 3:** The functional role of autophagy in mammalian systems.

Organs		Functions	Reviewed in References
**Reproductive system**	**Male**	- Spermatogonial proliferation & differentiation - Spermiogenesis - Spermiation - Testosterone synthesis - Acrosome biogenesis - Flagella biogenesis - Sperm motility - Modulates ectoplasmic specialization assembly - Degrades & clears the androgen-binding protein - Regulates tubulobular complexes distribution - Maintains normal cytoskeletal organization	[102,103,104,105]
	**Female**	- Corpus luteum regression - Promotes progesterone synthesis - Follicular growth & differentiation - Follicular atresia - Placentation - Endometrial remodeling - Oogenesis & embryogenesis - Promotes oocyte maturation & longevity - Early embryonic development - Eliminates paternal mitochondria	[106,107,108]
**Digestive system**	**Stomach**	- Regulates gastric mucosal cells	[109]
	**Intestine**	- Maintains barrier integrity - Preserves intestinal homeostasis - Regulates the function of Paneth cells - Prevents invasion of pathogens - Maintains mucosal immune response	[110]
	**Liver**	- Energy homeostasis of hepatocytes - Regulates gluconeogenesis & glycogen storage - Prevents hepatocellular degeneration - Degrades lipid droplets - Suppress hepatic tumors - Release of hepatokines	[111,112]
	**Pancreas**	- β-cell adaptation to high-fat diet - Maintains pancreatic β-cell mass - Regulates insulin content - Prevents trypsine autoactivation	[113,114]
**Brain**		-Regulates food intake & energy balance - Controls axonal integrity - Neuroprotective effect on neurological diseases	[115,116]
**Heart**		- Regulates cardiac homeostasis & function - Preserves cardiac structure - Mediates cardiac adaptation to pressure overload - Controls angiogenesis - Prevents age-related dysfunction	[117,118]
**Kidney**		- Maintains podocyte integrity - Maintains proximal tubule cell homeostasis - Protects against ischemic injury	[21,119]
**Lung**		-Regulates the airway’s responsiveness	[31]
**Immune system**		- Regulates cytokine production - Development of T and B cells	[120]
**Adipose tissue**		-Adipogenesis/Adipocyte Differentiation	[121]
**Bone**		- Regulates bone formation & resorption - Maintains osteocyte homeostasis - Differentiates osteoblasts & osteoclasts	[122]
**Skeletal Muscle**		- Maintains muscle mass & myofiber integrity - Preserves skeletal muscle function during aging - Release of myokines	[123]

## Data Availability

Not applicable.
